# The Emerging Trend of Bio-Engineering Approaches for Microbial Nanomaterial Synthesis and Its Applications

**DOI:** 10.3389/fmicb.2021.638003

**Published:** 2021-03-16

**Authors:** Raunak Dhanker, Touseef Hussain, Priyanka Tyagi, Kawal Jeet Singh, Shashank S. Kamble

**Affiliations:** ^1^Department of Basic and Applied Sciences, School of Engineering and Sciences, GD Goenka University, Gurugram, India; ^2^Department of Botany, Faculty of Life Sciences, Aligarh Muslim University, Aligarh, India; ^3^Amity Institute of Biotechnology, Amity University, Noida, India

**Keywords:** nanoparticles, genetic engineering, bacteria, viruses, bacteriophages

## Abstract

Micro-organisms colonized the world before the multi-cellular organisms evolved. With the advent of microscopy, their existence became evident to the mankind and also the vast processes they regulate, that are in direct interest of the human beings. One such process that intrigued the researchers is the ability to grow in presence of toxic metals. The process seemed to be simple with the metal ions being sequestrated into the inclusion bodies or cell surfaces enabling the conversion into nontoxic nanostructures. However, the discovery of genome sequencing techniques highlighted the genetic makeup of these microbes as a quintessential aspect of these phenomena. The findings of metal resistance genes (MRG) in these microbes showed a rather complex regulation of these processes. Since most of these MRGs are plasmid encoded they can be transferred horizontally. With the discovery of nanoparticles and their many applications from polymer chemistry to drug delivery, the demand for innovative techniques of nanoparticle synthesis increased dramatically. It is now established that microbial synthesis of nanoparticles provides numerous advantages over the existing chemical methods. However, it is the explicit use of biotechnology, molecular biology, metabolic engineering, synthetic biology, and genetic engineering tools that revolutionized the world of microbial nanotechnology. Detailed study of the micro and even nanolevel assembly of microbial life also intrigued biologists and engineers to generate molecular motors that mimic bacterial flagellar motor. In this review, we highlight the importance and tremendous hidden potential of bio-engineering tools in exploiting the area of microbial nanoparticle synthesis. We also highlight the application oriented specific modulations that can be done in the stages involved in the synthesis of these nanoparticles. Finally, the role of these nanoparticles in the natural ecosystem is also addressed.

## Introduction

Nanotechnology is the branch of science that supports the designing and manipulation of organic and inorganic matter to the nanoscale levels (1–100 nm; Hussain and Hussain, 2015; Danish and Hussain, 2019). A large variety of nanoparticles (NPs) have found place in different sectors of the economy. However, these NPs are produced under extreme physico-chemical conditions, which pose a threat to the environment. Therefore, in order to prevent the associated toxicity, the green approaches for NPs synthesis using biological systems are emphasized.

**Figure 1:**
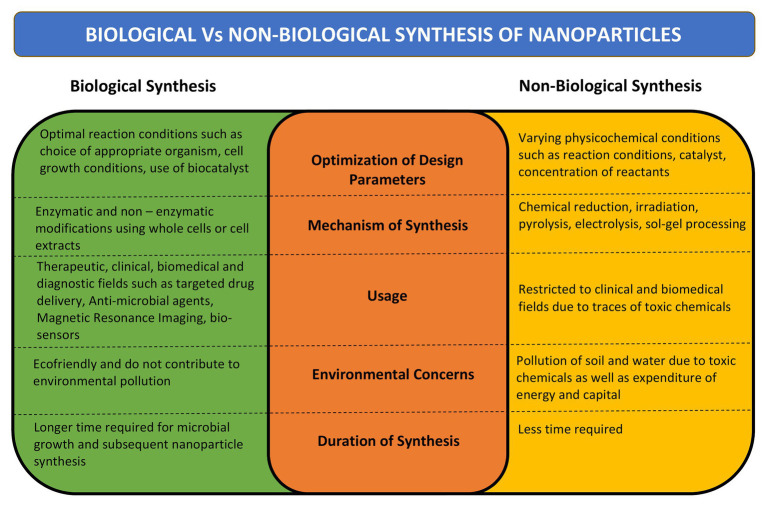
Comparison between biological and non-biological methods of nanoparticle (NP) synthesis. Comparison has been done on the basis of mechanism of synthesis, design parameters, time required for synthesis, its desired uses, and effect on environment.

The microbial bioprocessing is recently explored as an attractive alternative to chemical and physical fabrication of NPs. Microbial synthesis of NPs is an integration of nanotechnology with microbial biotechnology. Bacteria, archaebacteria, fungi, yeast, molds, microalgae, and viruses are being explored for synthesis of bioactive nanostructures with numerous industrial applications (Hulkoti and Taranath, 2014). The NPs produced by microbial biosynthesis and bioprocessing are mostly sustainable, eco-friendly, and cost-effective. However, the process of biosynthesis is time consuming and it is difficult to control the shape, size, and dispersity of the NPs. Several strategies have come up to overcome these limitations such as appropriate strain selection, development of genetically engineered microbes, optimization of microbial cultivation and extraction techniques, and combination approaches such as photo-biological methods (Bao et al., 2003; Mohammadian et al., 2007). A comparison between biological and non-biological synthesis of NPs is presented in Figure 1.

Specific microbial interactions with their surroundings lead to the production of bioactive NPs. The microbes residing in industrial and mining effluents have been found to recover metals from the discharged waste and converting them into nanostructures. Similarly, metal chelation in the form of NPs represents antagonistic interactions between rhizosphere associated microbes and pathogenic microbes (Gouda et al., 2019). The marine microbes are capable of forming exotic nanostructures by effective internalization of minerals from rich salty water by reduction/precipitation of toxic metal ions into non-toxic metallic nanoclusters to cause metal detoxification. The microbes are regarded as highly efficient eco-friendly nanofactories (Pimprikar et al., 2009; Li et al., 2011; Senapati et al., 2012; Gnanamoorthy et al., 2014; Manivasagan et al., 2016; Sánchez-López et al., 2020).

**Figure 2:**
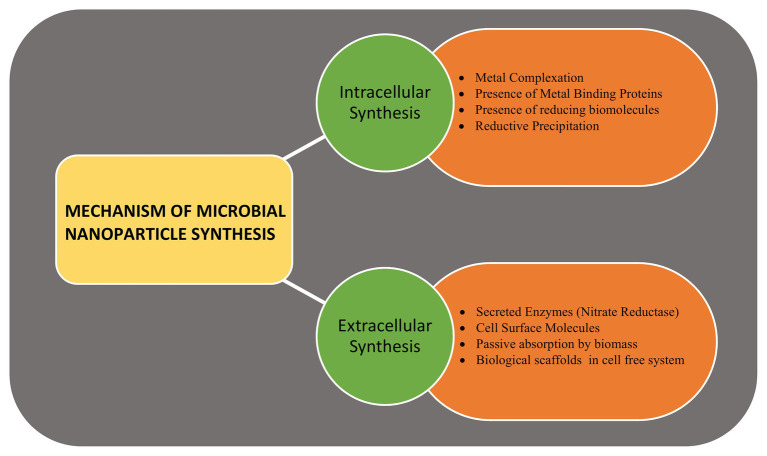
The mechanism of microbial nanoparticle synthesis. The intracellular synthesis includes various enzymes, reducing molecules, chelating agents, and metal binding proteins present within the cell, while the extracellular synthesis involves secreted enzymes, surface enzymes, proteins, and reducing agents.

Metallic, non-metallic, and metal-oxide NPs are produced by the microbes, intracellularly or extracellularly, *via* enzymatic reduction process (Manivasagan et al., 2016). Marine bacteria, fungi, and microalgae have been found to synthesize metalic NPs by intracellular or extracellular pathways. The mechanism of microbial synthesis of NPs involves the reduction of positively charged metal ions trapped in the cytoplasm or on the cell wall to fine nuclei by the negatively charged biomolecules that helps in forming the nanostructures (Golinska et al., 2014). Several fungal and algal species have been observed to produce gold (Au) and silver (Ag) NPs when exposed to metal salts in the external medium. Around 38 species of brown algae, 23 species of red algae, 49 species of green algae, and 21 species of blue-green algae are known to produce nanoparticles with wide variety of applications (Chaudhary et al., 2020). Similarly, around 30 species of fungi producing Au and Ag nanoparticles have been observed (Khan et al., 2017). The site of NPs synthesis is specific to the species and may be produced intracellularly either on the surface of mycelia/plasma membrane (such as *Rhodococcus* spp., *Tetraselmis kochinensis*) or below the cell wall surface (*Verticillium* spp.; Mukherjee et al., 2001; Ahmad et al., 2003). Similarly, the extracellular synthesis of metallic nanoclusters involves the role of surface proteins or enzyme secretion. The role of extracellular secretion of nicotinamide adenine dinucleotide phosphate α-NADPH dependent nitrate reductase enzyme is well-documented in case of fungus *Fusarium oxysporum* and bacteria *Rhodopseudomonas* (Khandel and Shahi, 2018). Extracellular biosynthesis of Ag NPs of size 10–15 nm produced by *Escherichia coli* VM1 has anti-cancer potential (Maharani et al., 2016), while the zinc sulphide (ZnS) NPs produced by *Desulforibrio caledoiensis*, has photo-catalytic applications (Qi et al., 2013). Recently, lignin peroxidase enzyme has been reported to be associated with the synthesis of Au and selenium (Se) NPs from *Acinetobacter* spp. SW30 (Wadhwani et al., 2018). The stable Se nanorods of size ~200 nm have been prepared using *Pseudomonas alcaliphila* (Zhang et al., 2011). Different species of *Cyanobacteria* such as *Spirulina*, *Anabaena*, and *Calothrix* form Au, Ag, platinum (Pt), and palladium (Pd) NPs of different size. Amorphous Se NPs (10–80 nm) are formed by *Nostoc linckia* by selenite reduction involving thiol groups of cyanobacterial proteins (Rai et al., 2019). Besides bacteria, the enzymes present on cell surfaces of yeasts and molds can efficiently synthesize monodispersed metal-oxide NPs with well-defined morphology. Zhou et al. (2009) synthesized mesoporous magnetite (Fe_3_O_4_) particles using yeast cells. *Saccharomyces cerevisae* have been found to produce ~20 nm antimony trioxide (Sb_2_O_3_) and titanium dioxide (TiO_2_) NPs. Other examples include zinc oxide (ZnO) NPs by *Aspergillus fumigatus* and *Candida albicans* (Zikalala et al., 2018). Microalgae have been reported to synthesize metal-oxide NPs by polysaccharides present in their cell walls. ZnO NPs are prepared by many species of algae such as *Sargassum muticum*, *Chlamydomonas* *reinhardtii*, and *Gracilaria gracilis* (Mahdavi et al., 2013). The mechanism of NP synthesis using microbial cells is shown in Figure 2.

**Figure 3:**
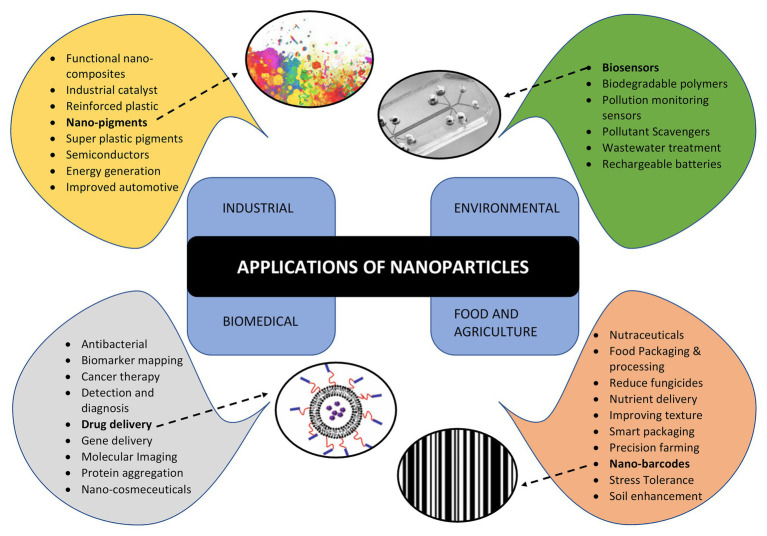
Applications of nanoparticles in various areas such as industries, environment conservation, biomedical diagnostics and therapeutics, food and agriculture highlighting various sub areas of each.

Apart from NPs, the microbes are also capable of synthesizing unique nanostructures. Recently, the biosynthesis of 1-D nanowires has generated considerable attention due to remarkable physico-chemical properties. Microbial nanowires refer to the filamentous appendages of metal-reducing bacteria in the genus *Geobacter*, which produces the conductive Type IV pili (T4P; Reguera, 2018). These nanowires play important role in the bioenergy strategies involving the transport of respiratory electrons to extracellular electron acceptors and, therefore, aids in species electron exchange (Malvankar et al., 2011; Kotloski and Gralnick, 2013). The microbial nanowire based electron transfer mechanism is one of the most significant systems in the microbial fuel cells (Lan et al., 2018), which are potential alternative to harvest energy from organic wastes by anaerobic digestion (Logan and Rabaey, 2012) and the nanodevices (Chen et al., 2010). This remarkable capacity of microbial nanowires is attributed to the assembly of micrometer-long polymers of the hexa-heme cytochrome OmcS in its nanowires (Wang et al., 2019). Bacterial biosynthesis of nanocellulose has opened up several avenues in biomedical research. It is present in the form of chains of 20–100 nm nanofibers in the exopolysachharides of bacterial cells (Klemm et al., 2011; Sivakumar et al., 2014). The critical issue of non-biodegradable nature of nanocellulose has been overcome by periodate oxidation to form dialdehyde nanocellulose (Li et al., 2009). The bio-cellulose derived from bacterial nanocellulose through culture cultivation finds broad applications in biomedical engineering such as stem cells/muscle cells based tissue engineering scaffolds (Lv et al., 2016), urethral reconstruction by nanocellulose seeded lingual keratinocytes (Huang et al., 2015), and antimicrobial wound dressings (Kim et al., 2015). The nanomaterials synthesized by microbes are not only environment friendly but also have a well-defined chemical composition, size, and morphology that makes them a promising candidate for potential applications such as drug delivery, bio- and environmental sensors, antimicrobial agents, and imaging. The applications of NPs synthesized by microbes are depicted in Figure 3.

However, there remains major drawback in large scale application of biological synthesis of nanomaterials such as heterogeneity in size, shape, and lesser control over the physicochemical properties. Genetic engineering in combination with molecular biology and biotechnology approaches provide a highly innovative and powerful tool toward the revolutionary path of tailor made and application oriented fabrication of various nanostructures. This review article highlights the recent advances in microbial synthesis of nanomaterials and the impact of bio-engineering approaches in directing tailor made synthesis of NPs for diverse applications. It also highlights the hidden potential of polymicrobial communities or biofilms in both synthesizing and stabilizing the NPs. Additionally, the review also highlights the use of less common microbes such as protozoa and archaea in the synthesis of NPs. Finally, the review emphasizes on the effect of these NPs on the ecosystem and the necessity of regulatory bodies to closely monitor the usage and disposal of such NPs from the industries and research laboratories. Thus, it provides a detailed compilation of various aspects of nanobiotechnology which has not been covered elsewhere.

## Bacterial Synthesis of NPs

Bacteria are prokaryotic microorganisms which are found in the different kinds of environmental conditions such as in the varying range of salinity, temperatures, alkaline, and acidic environments. They are unicellular, forming 50% of the biomass of the aquatic habitats. NPs synthesized using bacteria have been implicated in the fabrication of new materials for biomedical and health care purposes (Schrofel et al., 2014; Fariq et al., 2017). This approach of NP synthesis is more reliable, eco-friendly, and non-toxic (Mohanpuria et al., 2008).

Magnetotactic bacteria are capable of synthesizing the magnetite nanocrystals *via* phospholipid membrane bound vesicles called magnetosomes (Li et al., 2007). The metal ions are transported into the magnetosome vesicles and reduced by the reductase enzymes on cell surface followed by transportation into the phospholipid membrane (Philipse and Maas, 2002). However, the number of such NPs is very limited. Therefore, the researchers use another strategy of inducing the microbes to synthesize different metal oxide NPs during bioremediation of metal ion toxicity (Jayaseelan et al., 2012). Bacteria-induced ZnO and TiO_2_ NPs have been synthesized using *Rhodococcus* spp. and *Lactobacillus* cell culture solution, respectively (Jha et al., 2009; Kundu et al., 2014). *Pseudomonas aeruginosa* SM1 has been found to synthesize NPs from various metal ions without the need for growth media, stabilizing agent, pH optimization, and presence of electron donor. This strain can synthesize the NPs at both intracellular locations [cobalt (CO) and lithium (Li)] and extracellular locations [silver, palladium, iron, rhodium, nickel, ruthenium, and platinum (Ag, Pd, Fe, Rh, Ni, Ru, and Pt)] in crystalline as well as amorphous state at room temperature (Srivastava and Constanti, 2012). Other researchers reported the intracellular synthesis of the microscopic Au, Ag, and Au-Ag alloy crystals when *Lactobacillus* strain is mixed with high concentrations of each metal ion. The bacteria produced these NPs intracellularly, and the cells were able to maintain their viability even after crystal growth. Transmission electron microscopy (TEM) was used to examine crystallites of 100–300 nm covering the periplasmic space of the bacteria (Nair and Pradeep, 2002).

**Figure 4:**
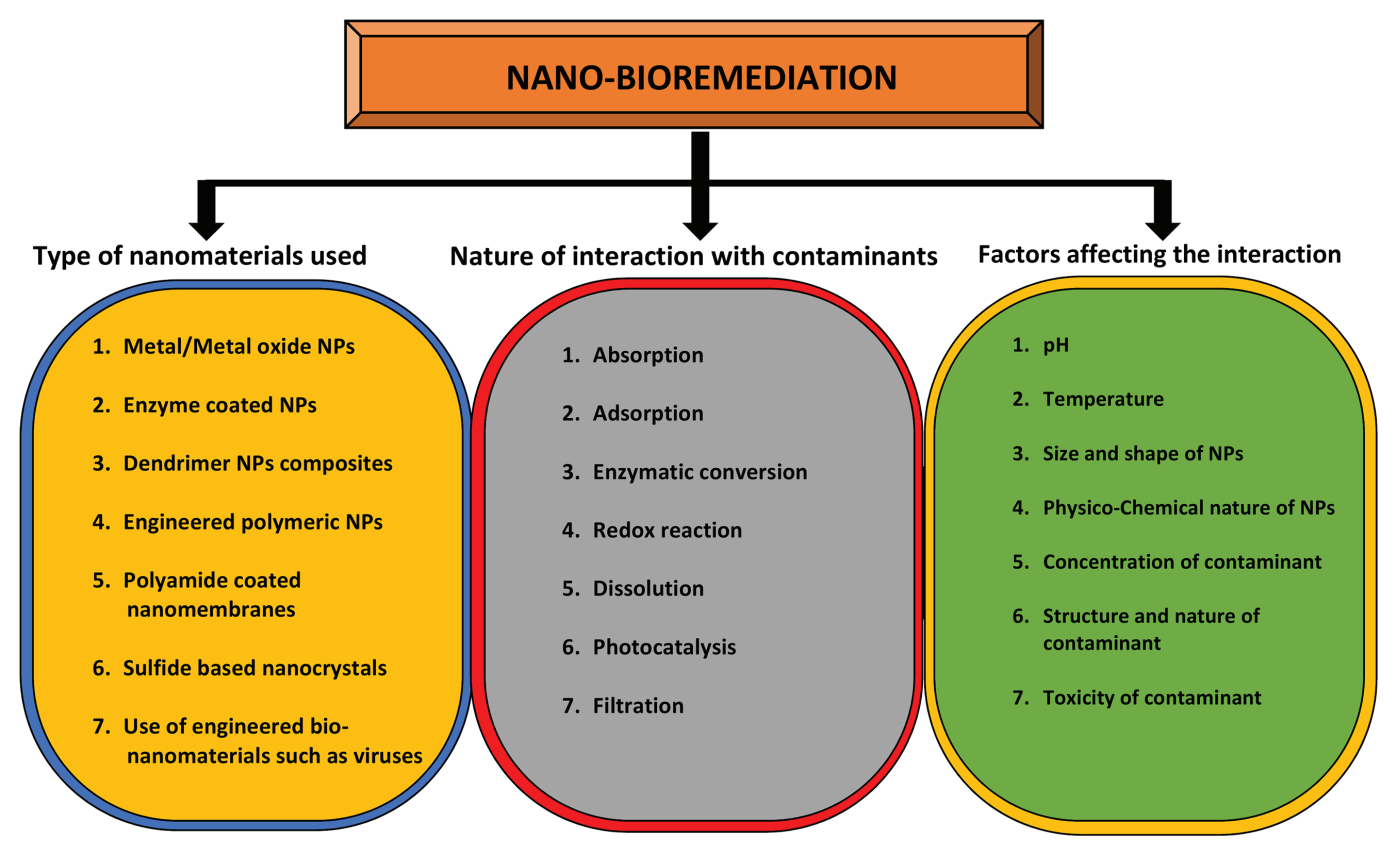
Role of nanomaterials in bioremediation and factors affecting it. The yellow circle indicates the types of nanomaterials that can be used for bioremediation purposes while the gray circle enlists the types of interactions between nanomaterials and contaminants. The outcome of this interaction depends on various factors as mentioned in green circle.

pH plays an important role in determining the size and shape of the NPs. The bacterium *Shewanella algae* was used for the production of Au NPs by using H_2_ as an electron donor under different pH conditions (Konishi et al., 2007). Au NPs of 10–20 nm were synthesized in the intermembrane space of *S. algae* cells at pH 7, whereas large sized NPs 50–500 nm were precipitated out of the cell. However, this species has also been reported as marine pathogen from squamous cell carcinoma patient (Sumathi et al., 2014). In a similar study, the bacteria *Rhodopseudomonas capsulate* was used to synthesize Au NPs of different shapes and sizes under a range of pH 4–7 (He et al., 2007). So, these studies indicate the pH specific control over the size and deposition locations of Au NPs. Spherical shaped NPs from 10 to 20 nm were formed at diluted concentration of tetrachloroaurate (AuCl_4_) at pH 6. However, Au nanowires were produced at increasing concentrations of AuCl_4_ at the constant pH. It was observed that when pH changed to 4, both triangular and spherical shaped NPs were formed at diluted concentrations of salt (Husseiny et al., 2007). The types of nanomaterials used for the purpose of bioremediation, the nature of interactions between NPs and metal(s), and the factors affecting these interactions are highlighted in Figure 4.

When two metals are combined together, they form a single bimetallic nanoparticle which provides the potential physical stability and exceptional magnetic, optical, and catalytic properties in comparison to single metallic particles. For instance, it is believed that if Pt is combined with a noble metal, it enhances the catalytic activity of the Pt group metal. Therefore, this integration is useful for different noble applications. However, very few evidences are available for such bimetallic NPs.

## Bioaccumulation of NPs Within Phytoplankton and its Role in Bioremediation

Phytoplankton are photosynthetic components of the aquatic grazer food chain. They are primary food source for zooplankton (Dhanker et al., 2012, 2013). However, chemicals secreted by many phytoplankton species for their self-defense play important role in shaping the population of grazers such as copepods (Dhanker et al., 2015). They can accumulate large levels of heavy metals inside them and can convert them into suitable nanostructures. This ability has found tremendous applications in the area of bioremediation that involves cleaning of polluted sites using various strategies. However, these NPs also cause serious damage to the phytoplankton depending on their concentration, size, shape, and type of metal involved. Therefore, suitable research approaches are needed to use algae for the purpose of bioremediation while keeping them intact and viable.

Algal mineralization has been recently explored in nanotechnology. Nanostructures have been synthesized either using live algal cells, cell free supernatant, or bioactive molecules extracted form cells. Ag NPs have been synthesized using live cells of algae belonging to Chlorophyta, Ochrophyta, Haptophyta, and *Spirulina* (Merin et al., 2010; Mohseniazar et al., 2011; Dahoumane et al., 2012; Rösken et al., 2014). This method has also been applied for the synthesis of bimetal Au-Ag alloy NPs (Dahoumane et al., 2014). In other study, cell free extract of *Euglena* spp. has been applied for the accumulation of Ag NPs (Li et al., 2015b). *Chlorella vulgaris*, when exposed to Ag salts for nearly 4 h, accumulated 1,200–3,300 μg/g dry weight of Ag NPs. Additionally, using the same system, *Raphiodocelis subcapitata* accumulated 45.0 μg/g dry weight of Ag NPs even after exposure for 24 h. This difference in absorption ability is due to exposure time, concentration of Ag ions, reducing conditions, and algal species involved (Ribeiro et al., 2015).

Members of the class Phaeophyceae, Chlorophyceae, Rhodophyceae, and Cyanophyceae have been widely explored for the synthesis of metallic NPs *via* intracellular or extracellular routes. Algae have rich source of antioxidants, pigments, terpenoids, amines, and alkaloids also known as the bioactive compounds that are shown to act as reducing agents favoring the synthesis of metal nanostructures (Asmathunisha and Kathiresan, 2013). The polyols and amides present in *C. vulgaris* have been shown to reduce the Pd salts to accumulate Pd NPs (Arsiya et al., 2017). Similarly, 10 different chemical constituents present in the extracts of *Galaxura elongata* such as glutamic acid, stearic acid among others has been shown to be involved in the synthesis of Au NPs (Abdel-Raouf et al., 2017). The siliceous diatoms have a large surface area and are readily functionalized for potential applications in enzyme immobilization (Gnanamoorthy et al., 2014; Kong et al., 2018). Cells of microalgae are interlocked by a shell of intertwined structures of calcium carbonate (CaCO_3_), derived from specialized intracellular vesicles and water-soluble acidic polysaccharides, called coccoliths. *Emiliania huxleyi*, is a dominant coccolithophore used as a source of mineralized material for nanotechnology applications such as nano-fluidics (Skeffington and Scheffel, 2018). The coccoliths are characterized by tightly controlled crystal growth and nucleation. Therefore, nanodevices constructed using coccoliths have potential applications in advanced electronics, environmental sensing systems, and drug delivery. Molecules may be selectively encapsulated in hollow funnel and tube shaped nanoscale pores of *Discosphaera tubifera*, *Pontosphaera japonica*, and *Michaelsarsia elegans* (Haywood et al., 2015).

The accumulation of NPs in algae depends on the initial concentration of NPs in the water. It is observed that the absorption process is much faster in the initial stage, but the absorption rate is gradually reduced as it reaches to saturation point (Harja et al., 2015). There is a significant difference in the amount of NPs absorbed by algae from water with a change of pH of the solution. Thus, the range of strong acidic pH increases the absorption possibilities of NPs by algae reasonably (Vijayaraghavan et al., 2011). Other factors are algae size, algal biomass, physicochemical properties, and dose of NPs which influence the absorption capacities of different algal species to NPs (Esmaeili and Beni, 2015).

Due to their capacity to accumulate large amounts of NPs, various types of algae are being used for the process of bioremediation. However, various studies showed that NPs interact with various cellular sites within the algae, to interrupt with their normal functioning. The interaction of NPs with plasma membrane disrupts the arrangement of its basic components and releases enzyme lactate dehydrogenase into the cell cytoplasm. This may be one of the cell death mechanisms *via* inducing oxidative stress response and disturbing the cell integrity (Bhuvaneshwari et al., 2015). The exposure to TiO_2_ NPs induced oxidative stress response pathway in *Anabaena variabilis* damaging the cell membrane (Cherchi et al., 2011). In another study, NPs were found to loosen the bonding between the cellular components and enhancing the membrane permeability, leading to the entry of NPs within the cells (Pal et al., 2007). Furthermore, the damaging effects of NPs have been observed on the chloroplast evident by the disruption of the thylakoid lamellae (Hu et al., 2014). Exposure to the nanotubes in high doses cause swelling in endoplasmic reticulum (Jia et al., 2005). NPs can affect the function of mitochondria which in turn influence the metabolic activities of algal cells (Zhao et al., 2016). Further, NPs cause clumping of the chromatin adjacent to nuclear membrane leading to nuclear dysfunctioning in *Acidophila* (Melegari et al., 2013; Bhuvaneshwari et al., 2015).

Production of reactive oxygen species (ROS) by NPs is another mechanism of toxicity as ROS oxidizes numerous biomolecules such as proteins, lipids, nucleic acids, and carbohydrates leading to loss of function (Larguinho et al., 2014). It also induces gene mutations and oxidative stress response inside the recipient cells while inhibiting cellular enzymatic activities (Melegari et al., 2013). The accumulation of NPs on the surface of algal cells leads to what is known as the shading effect, which perturbs the light absorption capacity of the photosynthetic apparatus (Perreault et al., 2012; Li et al., 2015a; Chen et al., 2018). Aluminium oxide (Al_2_O_3_) NPs have been found to reduce the content of chlorophyll in *Chlorella* spp. and *Scenedesmus* spp. (Sadiq et al., 2011). It has also been demonstrated that silicon dioxide (SiO_2_) NPs affect the photosynthesis process in *Scenedesmus* spp., by reducing the content of chlorophyll a and b without affecting the carotenoid content (Wei et al., 2010).

## Mycogenic Synthesis of NPs

Fungi are the eukaryotic heterotrophs with a typical decomposing food habit. They can be unicellular such as yeasts which are shown to synthesize NPs with semiconducting property (Roy et al., 2015; Venkat-Kumar et al., 2019). The multicellular fungi are known as molds which produce filamentous hyphae. The increasing role of fungi in nanobiotechnology has attracted the worldwide attention to synthesize green metallic NPs (Alghuthaymi et al., 2015; Moghaddam et al., 2015). Mycogenic synthesis of NPs is generally preferable due to advantages such as easy handling, less nutritional demands, huge biomass production, better yield, high tolerance, and non-pathogenicity for human use (Dar et al., 2013; Ahluwalia et al., 2014). Extracellular production of fungal metallic NPs has been studied in detail in the last few decades (Castro-Longoria et al., 2011; Devi and Joshi, 2012; Castro et al., 2014; Mishra et al., 2014). Many yeast species have been found to synthesize Ag NPs having antifungal activity (Calvo et al., 2010; Eugenio et al., 2016). The Ag-Au alloy NPs produced by yeast cells have shown promising application in the synthesis of electrochemical sensors for the detection of paracetamol and vanillin (Zheng et al., 2010; Wei, 2017). The Pd NPs synthesized using the extracts of *S. cerevisiae* are shown to be useful in the photo catalytic degradation of azo dye used in textile industries (Sriramulu and Sumathi, 2018). The nitrate reductase enzyme produced by certain yeast and fungi species have been reported for the production of NPs. *Fusarium oxysporium* strains produce Ag NPs by reduction of Ag^+^ ions, using species-specific nitrate reductase enzymes (Duran et al., 2005). Fernandez et al. (2016) utilized the nitrate reductase activity present in the cell free supernatant of the culture of *Cryptococcus laurentii* and *Rhodotorula glutinis* to synthesize Ag NPs. They found a direct correlation between the concentration of NPs and the nitrate reductase enzyme activity.

The cell wall of fungi plays an important role in the reduction of metal ions during intracellular production of NPs (Sastry et al., 2003). In brief, the metal ions present in the medium are trapped on the fungal cell surface due to electrostatic attraction by the wall enzymes, followed by the enzymatic reduction leading to the formation of NPs. The psychrotrophic marine strain of the *Yarrowia lipolytica* was analyzed for the production of cell-associated Ag and Au NPs in the presence of metallic salts (Agnihotri et al., 2009). However, the extraction of cell-associated NPs becomes difficult. Therefore, the cell associated biopolymer melanin was isolated from the cell free culture of *Y. lipolytica* and employed for the synthesis of highly stable free form of Ag NPs having antibacterial activity (Apte et al., 2013). The proteins present in the fungal extract get adsorbed on the surface, thereby enhancing the colloidal stability of the Ag NPs (Du et al., 2015). Ag NPs synthesized by *R. glutinis* and *Rhodotorula mucilaginosa* were found effective in degrading the highly toxic chemical pollutants such as methylene blue (MB) and 4-nitrophenol (4-NP; Vidhu and Philip, 2014; Cunha et al., 2018).

The recent advancement in biological tools and techniques has been used by the researchers for the synthesis of improved metallic NPs. The engineered fungal and yeast cells prove to be more advantageous compared to the conventional physicochemical methods being eco-friendly, less toxic, cost effective, and production of monodisperse NPs. Recombinant fungi and yeast cells have been utilized for the biosynthesis of intracellular metallic NPs that are highly dispersed, stable, and safe (Siddiqi and Husen, 2016). Genetically modified metal resistant *Pichia pastoris* exhibiting the overexpression of *Mucor racemosus* cytochrome b5 reductase enzyme (Cyb5R) was successfully used as a reduction system for the synthesis and biosorption of intracellular Ag and Se nanoparticles (Elahian et al., 2017).

## Protozoans as Cellular Factories for Nanomaterial Synthesis

In recent times, synthesis of NPs utilizing microbes have been explored to a great extent but little is known about the use of protozoans as bio factories. Very few studies were reported so far showing the potential of protozoa in synthesizing metallic NPs. For instance, *Leishmania* sp. had been used for the synthesis of Ag and Au NPs. These NPs were found to be within the range of 10–100 and 50–100 nm scale, respectively (Ramezani et al., 2012). Currently, Ag NPs are being synthesized utilizing *Pseudomonas* strain related with the antarctic psychrophilic protozoon *Euplotes focardii* (John et al., 2020). In another study, *Tetrahymena thermophila* SB210 was used for *in vivo* synthesis of nano-Se within the range of 50–500 nm scale (Cui et al., 2016). Recently, calcite skeletal structures of marine protozoa foraminifera have been used for the synthesis of magnetic nanocomposites. This is, the first report of bionic synthesis of nanocomposites using the natural biomineralization pathway (Magnabosco et al., 2019). Other studies have reported the biosynthesis of QDs by *Tetrahymena pyriformis*. The synthesized nanomaterial was found to be around 8.27 nm in diameter and emitted yellow fluorescence, characteristic of metal ion Cd^2+^ (Cui et al., 2019). Furthermore, cell-free exudates of the ciliated protozoan *T. thermophila* were used to convert silver nitrate (AgNO_3_) to Ag NPs under illumination with fluorescent tubes at ambient temperature (Juganson et al., 2013).

## Archaea for the Synthesis of NPs

Archaea are the single celled prokaryotes inhabiting in a broader range of habitats. They include both extremophiles and non-extremophiles living in moderate conditions as well as in presence of extremes of pH, temperature, and salt concentration. Thus, they inhabit various places such as soil, deep oceans, marshlands, animal intestine, hydrothermal vents, hot water springs, and dead sea (DeLong and Pace, 2001).

Heavy metal tolerance has been exhibited by many archaeal species such as *Sulfolobus Solfataricus* (Schelert et al., 2004, 2006), *Thermoplasma acidophilum* (Ruepp et al., 2000), *Ferroplasma acidarmanus* (Baker-Austin et al., 2007), and *Halobacterium* sp. (Ng et al., 2000). However, many of the extremophilic archaeal species are difficult to grow in laboratory due to limitations in mimicking the natural conditions in which they live. Recently, two halophilic archaea, *Haloferax* sp. and *Halogeometricum* sp. isolated from solar saltern have been shown to synthesize Ag and Se NPs, respectively. The mechanism of NP synthesis involves both intracellular and extracellular reduction of metal ions. These NPs show remarkable uniformity in size indicated by polydispersity indices and have antibacterial activity against variety of pathogens (Abdollahnia et al., 2020). In another study, *Halococcus salifodinae* BK3, a haloarchaea was used for the synthesis of Ag NPs using AgNO_3_ salts. This intracellular mode of NP synthesis showed the role of NADPH-dependent nitrate reductase in metal tolerance, its reduction and synthesis of NPs (Srivastava et al., 2013). The similar group later showed the synthesis of Se NPs from sodium selenite (Na_2_SeO_3_) using another isolate *H. salifodinae* BK18. The mechanism of NP synthesis is similar to the one involved in the synthesis of Ag NPs using BK3 isolate (Srivastava et al., 2014). Another study involved the use of *Haloferax volcanii* in the synthesis of Au and Ag NPs with better antibacterial and catalytic properties (Costa et al., 2020). *Metallosphaera sedula*, an extreme thermoacidophilic archaeon which oxidizes metals during respiration have been used to synthesize tungsten nanostructures. These nanoscale metallo-organic complexes are formed between archaeal cells and tungsten polyoxometalate on which the archaea are grown. This process is accompanied with the accumulation of intracellular tungsten NPs consisting of cluster of atoms (Milojevic et al., 2019). The hyperthermophilic archaea, Thermococcales living in the hydrothermal deep sea vents produce Fe_3_S_4_ nanocrystals extracellularly using iron phosphate as the precursor. This process implicates a mechanism of carbon dioxide (CO_2_) homeostasis in hydrothermal ecosystems as greigite is the major catalysts for CO_2_ reduction (Gorlas et al., 2018). Another group of researchers used thermos-acidophilic archaea, *Sulfolobus tokadaii* for the reduction of Pd (II) to Pd (0) NPs in a redox reaction (Kitjanukit et al., 2019). In a latest study, mass scale production of superparamagnetic iron oxide (Fe_2_O_3_) NPs has been achieved using *Halobiforma* sp. N1 with better properties suited for localized hyperthermia therapy used in cancer treatment (Salem et al., 2021).

The self-assembly of the archaeal surface layer (S-layer) has been an area of extensive research. Using the S-layer from *Sulfolobus acidocaldarius* as a template, Pt NPs encapsulated within the dendrimers can be synthesized with excellent topochemical properties (Mark et al., 2006). The ferritin protein system of *Pyrococcus furiosus*, a hyperthermophilic archaeon encapsulates Ag NPs and have rather specific binding and nucleation sites for Ag(I), not observed in ferritin templates from other microbial systems (Kasyutich et al., 2010). The same ferritin template from *P. furiosus* has been recently implicated in the synthesis of Au and Pd NPs with detergent modified enzymatic activities (Peskova et al., 2019). This protein based template represents an excellent platform for the bio fabrication of metallic NPs. In a rather interesting approach, the cells of *S. acidocaldarius* possessing only the outermost S-layer, termed as the cell ghosts, were successfully used for the fabrication of Au NPs. These NPs consisted exclusively of Au (0) NPs rather than a mixture of different oxidation states of Au metal with unusually strong paramagnetic properties (Selenska-Pobell et al., 2011).

## Viruses as Self-Assembling Nanoconstructs

The ability of biological molecules such as proteins, viruses, and DNA to self-assemble in a solution is a promising approach for predesigned engineering nanostructures. These engineered nanostructures self-assemble to form functional nanoconstructs and are used in biomedicine field for the targeted drug delivery, vaccine preparation, gene therapy, bioimaging, tissue engineering, and specific cell targeting (Keum et al., 2011; Wu et al., 2013).

Viruses are nanosized entities consisting of a capsid, a protein shell enclosing a viral genome (Roos et al., 2010). They represent an intracellular self-assembling nanoconstruct and, therefore, have the potential to be used for the synthesis of engineered NPs. Several virus encoded proteins form stable nanoparticle configurations that self-assemble in infected host cells to wrap up the viral genetic material as a pre-imperative for propagation. However, non-replicating and non-infectious self-assembled viral nanoconstructs as prefabricated nano-scaffolds have been formed if scaffold proteins are assembled in the absence of viral genome (Lopez-Sagaseta et al., 2016).

This property of viral capsid to self-assemble around nucleic acid under physiological conditions, allows for alteration and modification in their structure to desirable nano-construct form. The basic principles involved in this process include alterations in the surface charge, electrostatic interactions, chemical conjugation, and covalent attachment by genetic manipulations (Pokorski and Steinmetz, 2011). The self-assembled viral nanoconstructs enter the host cell, multiply there by efficiently delivering their genetic material using the host intracellular machinery to produce progeny viruses. This property of virus allows for their use in the medical fields such as gene therapy but pathogenicity of animal viruses limits their use (Makkonen et al., 2015). Interestingly, bacteriophages and plant viruses such as tobacco mosaic virus (TMV), cowpea chlorotic mottle virus (CCMV), red clover necrotic mosaic virus (RCNMV), bacteriophage MS2, and *Salmonella typhimuriam* bacteriophage P22 are being focused more as they are safe to be used in humans (Wu et al., 2013).

Over the past decades, advancements in nanotechnology have allowed for the fabrication of nano-scale devices and their utilization in the biomedical field (Li and Wang, 2014; Lee et al., 2016). Viruses as self-assembling nanoconstructs open up the new opportunities for the development of energy storage devices, biosensors, and drug delivery systems. They allow target-specific drug delivery by encapsulating DNA/RNA, antigens, drugs, and enzymes. More recently, viral nanoconstructs have been used to develop light-harvesting systems (Yokoi et al., 2010). Furthermore, viral nanoconstructs enable target-specific delivery of antigens and amplification of the immune responses. Several structural proteins derived from different viruses served as the templates for nanoconstructs to deliver vaccine candidates such as core and surface antigens of Hepatitis B Virus (HBV), *Human Parvovirus* B19, *Papillomavirus*, *Bluetongue Virus*, TMV, *Picorna Virus*, etc. These subunit vaccine candidates were designed in such a way that they potentially mimic structural repetitiveness of the natural host-pathogen surface interactions, thereby providing improved antigen stability and immunogenicity. Several preclinical vaccine trials based on this strategy may be applicable against infectious diseases such as influenza, malaria, hepatitis, rabies, and AIDS (Lopez-Sagaseta et al., 2016).

Other group of researchers illustrated the precise self-assembly of NPs into ordered nanostructures directed by TMV coat protein (Zhang et al., 2019). Matsuura et al. (2020) prepared ribonuclease-decorated artificial virus-like capsid by the self-assembly of β-annulus-S-peptide. These constructs were created by the interaction between S-peptide moiety and S-protein. The recombinant TMV coat protein monomers with a reactive cysteine residue were developed to attach three thiol-reactive chromophores to viral structure and an efficient energy transfer was observed and recorded using fluorescence spectroscopy (Miller et al., 2007). Besides, bacteriophage MS2 contains translational repression (TR) operator protein that binds to its RNA stem-loop. Modification of the protein with the drug such as ricin A and 5-fluorouridine allows the protein to diffuse inside the viral nanoconstructs and bind steadily to the capsid to achieve targeted therapy (Wu et al., 1995). Recently, bacteriophage M13 and its relatives are mostly used to design genetically engineered viruses, which find their place in the development of phage-based nanosensors, fabrication of nanomaterial, nanofibers, and tissue regeneration (Pires et al., 2016). The recombinant phage tail sheath protein, gp053 isolated from *E. coli* infects *Myovirus* vB_EcoM_FV3 (FV3) and self-assemble to form stable polysheaths with potential applications in different fields of nanosciences (Šimoliunas et al., 2019). An optical nano-construct was designed by deleting the genome of Brome Mosaic Virus (BMV) and doping with indocyanine green (ICG), an FDA approved near-infrared (NIR) chromophore that allows site specific deep tissue optical imaging (Jung et al., 2011).

Although, engineering of the self-assembled nanoconstructs is a challenging task. The different strategies involved in nanoconstruct manipulation involve rational construction through conventional recombinant DNA technologies and microbial protein production. C3-symmetric molecular design of peptides and their characteristic self-assembly into virus-like nanostructures is a novel strategy. Through this strategy, trigonal conjugates of b-sheet-forming peptides, trigonal conjugates of glutathione, and a viral b-annulus peptide fragment were formed (Matsuura, 2012). However, these assemblies involve weak interactions. Thus, measurements of the self-assembly kinetics of individual viral capsids around their RNA genome was identified by using interferometric scattering microscopy. The results indicated that the self-assembly proceeds by nucleation, followed by monotonic growth (Garmann et al., 2019). Beside, nanoconstruct designing depends on the physical principles of virus assembly related to packaging, encapsulation, and capsid modification. Furthermore, several factors, such as pH, charge on capsid protein, and the effect of amino acids, also play an important role (Stella and Turvilee, 2018; Chaudhary and Yadav, 2019).

## Synthesis of NPs by Polymicrobial Communities

Most of the microbes in their natural habitats do not exists in pure culture and are bound to get surrounded by other types of micro-organisms. In this scenario, these microbes must adapt themselves to co-inhabit with others to form communities that are referred to as “polymicrobial communities.” From time to time, these communities undergo dynamic changes in terms of the proportion of the individual microbial type due to the intrinsic and extrinsic factors. However, it is the net outcome of this “collaboration” that determines the longevity of these communities (Kim et al., 2020).

The complex interplay between the co-habiting partners of these communities is very intriguing. Many times, this crosstalk is facilitated through secretion of various bioactive molecules either directly into the extracellular milieu or *via* membrane vesicles. Majority of infectious diseases involve the formation of polymicrobial communities that are difficult to eradicate completely with the help of current treatment regime. The polymicrobial nature of biofilms underlying the pathogenesis of cystic fibrosis has been revealed using an integrated approach of morphological, biochemical, and molecular methods. Using a combination of multiple techniques such as plate count, q-PCR, and fluorescent *in situ* hybridization using peptide nucleic acid probes (PNA-FISH), it was observed that these biofilms consists of a combination of *P. aeruginosa*, *Inquilinus limosus*, and *Dolosigranulum pigrum*. The combination of these species within the biofilms varied under stresses of oxygen concentration and presence of antibiotics (Lopes et al., 2018). Such kind of communities can also exist on artificial solid supports such as catheters, pacemakers, etc. In another study, the biofilms of mine drainage systems precipitating ZnS was found to be consisting mainly of sulfate reducing bacteria belonging to the family Desulfobacteriaceae along with other genera including the *Cytophaga/Flexibacter/Bacteroides* (CFB group), *Planctomycetales*, *Spirochaetales*, *Clostridia*, and green non-sulfur bacteria. The bacteria belonging to family Desulfobacteriaceae predominated the older and mature biofilms that are ZnS rich, whereas fresh and ZnS poor biofilms have a more diverse proportion of individual communities (Labrenz et al., 2000). Thus, it is essential to understand the complex dynamics of these polymicrobial communities for better control over them. These tiny communities need special attention from the clinical point of view, at the same time, they can be exploited for beneficial purposes.

Majority of these polymicrobial communities exists in the form of biofilms. A biofilm is a three-dimensional (3D) niche in which single or multiple bacterial species can co-exist within the framework formed by the extra cellular polysaccharides (EPS) secreted by its habitants. EPS can consist of xanthan, dextran, succinoglycan, hyaluronic acid, alginate, and several other polymeric substances, depending on the type of microbes involved in biofilm formation. Xanthan gum is produced by *Myxococcus xanthus*, whereas dextran is produced by *Leuconostoc*, *Lactobacillus*, and *Streptococcus* genera. Gellan and curdlan based EPS are produced by *Sphingomonas* spp. and *Alcaligenes faecalis*. Cellulose based EPS is a characteristic of *Agrobacterium*, *Azotobacter*, *Rhizobium*, *Salmonella*, etc., while Levan is a major EPS component in the biofilms of *Mycobacterium*, *Pseudomonas*, *Corynebacterium*, and *Bacillus*. Succinoglycan is another type of polysaccharide observed within the biofilms of *Alcaligenes*, *Pseudomonas*, and *Agrobacterium* (Glenn et al., 2007; Flemming and Wingender, 2010; Freitas et al., 2011; Donot et al., 2012; Mishra and Jha, 2013; Casettari et al., 2015; Habibi and Khosravi-Darani, 2017; Moussa et al., 2017). Recent types of bacterial EPS are FucoPol and GalactoPol with the former being produced by Enterobacter A47 and the later by *Pseudomonas oleovorans* aNRRL B-14682. Both these EPSs are formed by their respective bacteria while using glycerol as the sole source of carbon (Freitas et al., 2009; Ferreira et al., 2014).

Biofilm provide a safer niche for its inhabitants due to its inherent resistance to antimicrobial agents, various mechanisms for neutralizing toxic metals from the surroundings, better protection from host immune responses and other hostile conditions that becomes difficult for planktonic bacteria to manage. The cells within the biofilms of *P. aeruginosa* were found between 2 and 600 times more resistant to the heavy metals such as copper (Cu), lead (Pb), and zinc (Zn) than free swimming cells (Teitzel and Parsek, 2003). The resistance to toxic metals and other antimicrobial agents is acquired through multiple ways such as sequestration of positively charged metals by negatively charged species in the biofilm EPS such as phosphate, sulfate, carboxyl groups etc. (Hunt, 1986). Additionally, majority of antimicrobial agents are effective against actively metabolizing cells and their efficacy is reduced against stationary cells. Since biofilm is a 3D structure with multiple cellular layers, it contains layers of metabolically active, intermediate, and dormant cells. These dormant cells often are found to persist within the deepest layers of biofilm where the nutrients and oxygen are in scarcity. These dormant cells are responsible for reforming the mature biofilm after the initial onslaught of antimicrobial agent and thus establish recurring infections (Spoering and Lewis, 2001). The proteome of EPS within the biofilms of *Shewanella* spp. have been shown to contain redox proteins that play essential role in the immobilization of uranium (U; Cao et al., 2011). The same findings have been reported by other research groups using various micro-organisms such as *Synechococcus elongates*, *Acidithiobacillus ferrooxidans*, *M. xanthus*, and *Pseudomonas stutzeri* DSM 5190 (Macaskie et al., 2000; Merroun et al., 2003; Jroundi et al., 2007; Merroun and Selenska-Pobell, 2008; Acharya et al., 2009). *E. coli* which is genetically engineered for enhanced EPS production has been found to possess greater resistance toward the toxicity of Ag NPs. The production of EPS in *E. coli* is controlled by capsule synthesis (cps) operon that is positively regulated by *rcsA* gene. *Escherichia coli* overexpressing *rcsA* cloned in pSB1A2 overproducing colonic acid based EPS was found to exhibit better resistance to the activity of Ag NPs. Addition of exogenous xanthan based EPS to bacteria that lacked *rcsA* also confers the similar advantage by increasing the aggregation of NPs and also reducing the surface area of bacterial cells exposed to NPs (Joshi et al., 2012). Although mainly consisting of polysaccharides, these biofilm EPS also contains many of essential organic and inorganic components which play crucial role in metal immobilization, thus proving to be a better alternative over commercially available polysaccharides.

Biofilms also provide some advantages for the production of NPs compared to planktonic bacteria such as larger surface area, higher biomass accumulations that allows for hassle free, efficient, economic, and large scale synthesis of NPs with minimal requirement for precursor molecules. The biological synthesis of NPs requires metal ions and the agent to reduce them to NPs. These biofilms provide an array of natural reducing molecules such as proteins, lipids, and other aromatic compounds which are essential for the nanoparticle synthesis. A biofilm of metal reducing bacteria such as *Geobacter sufurreducens* have been shown to provide a better reducing environment for metal ions and thus are efficient in synthesizing NPs than their planktonic counterparts. Also, the rate of extracellular reduction of Pd (II) to Pd (0) NPs was found to be affected by the nature of the electron donor and was significantly enhanced by the addition of external reducing agents (Pat-Espadas et al., 2013a,b; Yates et al., 2013). Thus, biofilms can be manipulated to have better efficiency in synthesizing NPs at various levels.

Sulfur reducing bacteria (SRB) has been used for many years for bioremediation of metal polluted soils and water bodies. Majority of studies involving SRB have been performed using *Shewanella* spp., *Desulfovibrio* spp., and *Geobacter* spp. (Ayangbenro et al., 2018). These bacteria can also be used for the bioremediation of toxic metals from sewage plants as these bacteria grow well on organic resources (De Lima et al., 2001). The naturally occurring biofilms of these bacteria can precipitate several toxic metals such as cadmium (Cd), Co, chromium (Cr), Cu, manganese (Mn), Ni, and Zn as insoluble sulfides (White et al., 1998). Synthesis of sphalerite (ZnS) NPs has been shown within the naturally occurring biofilms of aerotolerant sulfate reducing bacteria from the family, *Desulfobactericiae*. The concentration of sphalerite within these biofilms is 10^6^ times higher than in the surrounding water indicating the predominant mechanism of controlling the metal levels in the groundwater and wetlands. These ZnS NPs can be as very fine in size ranging from 2 to 5 nm and also accumulates 0.01% by weight of arsenic (As) and 0.004% by weight of Se (Labrenz and Banfield, 2004). Thus, biofilms provide safe and inert niche for the nanoparticle synthesis in which the rate of synthesis can be controlled.

**Figure 5:**
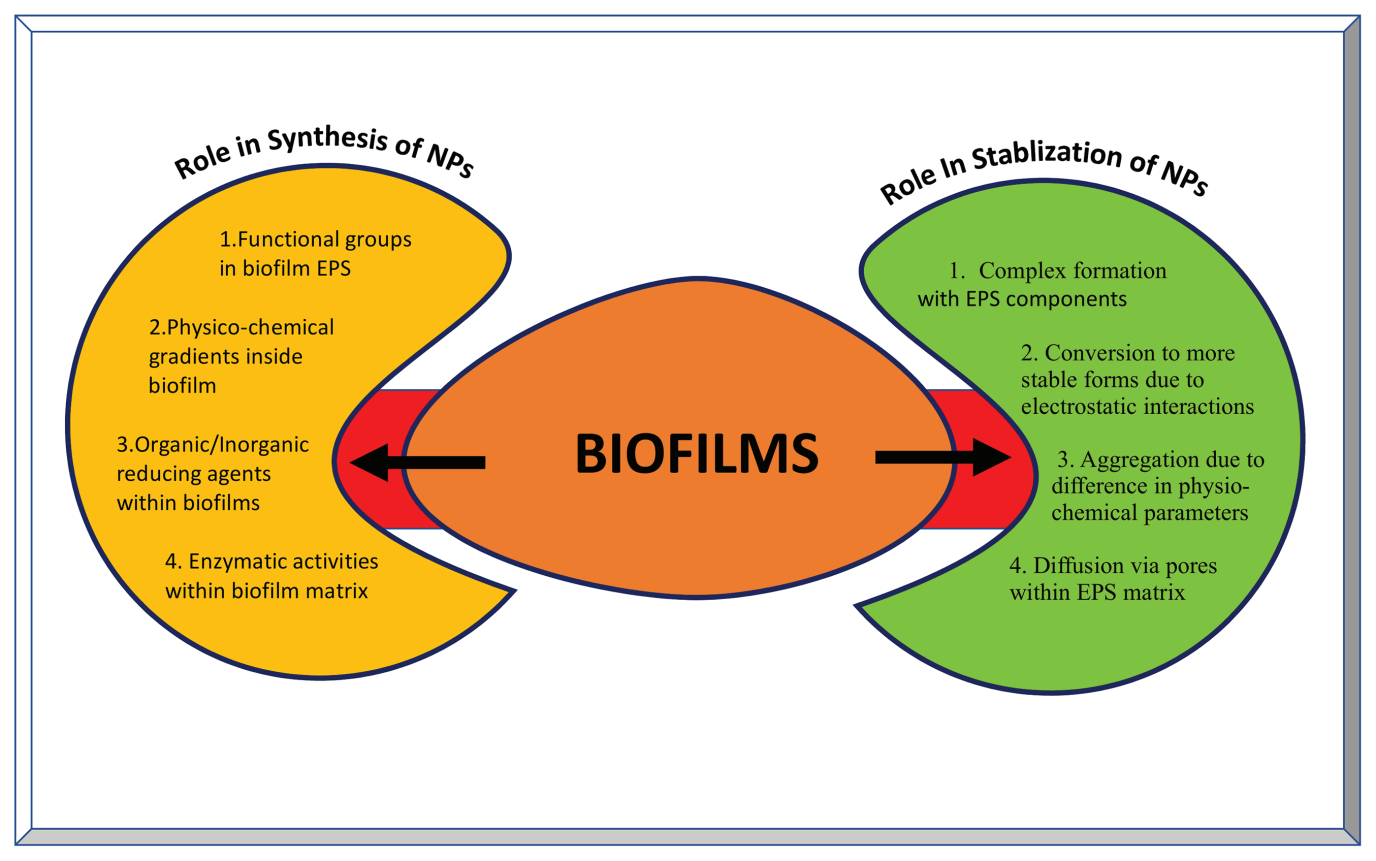
Role of biofilms in the synthesis and stabilization of nanoparticles. The left yellow panel indicates various factors contributing to the NP synthesis inside biofilms, while the right green panel indicates various outcomes of NPs-biofilm interaction leading to stabilization of NPs..

Recently, EPS purified from such biofilms has been used for the synthesis of metal NPs having wide variety of applications. Xanthan gum is effective in the synthesis of Ag and Au NPs with better catalytic and antibacterial properties. It also acts as drug carrier for doxorubicin hydrochloride against human lung cancer cell lines (Xu et al., 2014). Au NPs stabilized using dextran sulfate have shown better antitumor effects against carcinoma in the mouse model and better bactericidal properties against many Gram-negative and few Gram-positive pathogens (Cakic et al., 2016). Moreover, Ag NPs coated with dextran polysaccharide isolated from *Leuconostoc mesenteroids* T3 have better sensitivity and selectivity toward detection of cysteine in aqueous solutions (Davidovic et al., 2017). Similarly, purified carboxylic curdlan has been used for stabilizing Se and Zn NPs having improved antibacterial and antitumor activity (Yan et al., 2018). A combination of gellan and alginate based EPS matrix can be used as a stabilizing and catalyst system for Pd NPs (Cacchi et al., 2012). This strategy of using a combination of various EPS materials can be applied for the synthesis of other NPs as well. Moreover, the EPS production can be tailor made using genetic, molecular, and other biotechnology tools. Succinoglycan, levan, and cellulose based EPS have been shown to act as reducing and/or stabilizing agents for the synthesis of metallic NPs (Galvez et al., 2018; Gonzalez-Escarcega et al., 2018). The means by which biofilms are crucial in synthesizing as well as stabilizing nanoparticles are depicted in Figure 5.

Overall, approximately 150 representative bacterial species have been used in various studies for synthesis of NPs, whereas in case of fungi, about 80 species have been used for this purpose (Sastry et al., 2003; Korbekandi et al., 2009; Li et al., 2011; Iravani, 2014; Kitching et al., 2015; Moghaddam et al., 2015; Siddiqi and Husen, 2016; Sehgal et al., 2018; Gahlawat and Choudhary, 2019; Grasso et al., 2019). Similarly, around 130 species of various classes of algae have been used for the nanomaterial synthesis (Khan et al., 2017; Chaudhary et al., 2020). While the majority of the research has been done bacteria, fungi, and yeasts, the hidden potential of protozoa and archaea for nanomaterial synthesis is yet to be explored as very few species from each of them have been used for the synthesis of NPs. In addition, microbial biofilms also have tremendous potential for the synthesis and stabilization of NPs.

## Impact of Bio-Engineering Approaches on Microbial Nanomaterial Synthesis

**Figure 6:**
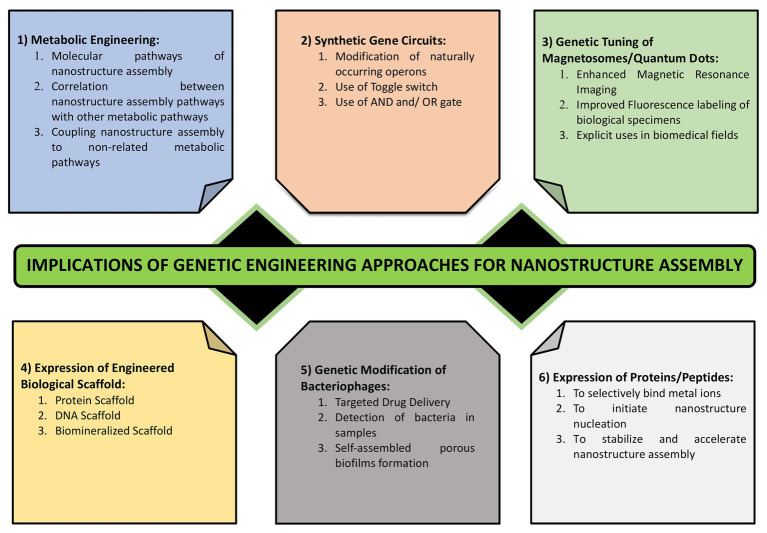
Various approaches for engineering the process of nanostructure synthesis and assembly. It highlights the importance of genetic tuning of cellular structures such as magnetosomes, metabolic engineering, metabolic coupling, expression of metal binding proteins and those involved in metal reduction and complexation, use of synthetic regulated gene circuits, and the use of cell free scaffolds.

In natural ecosystem, microbes encounter numerous metals in close vicinity. Some of them die owing to the toxic nature of these metals while others persist which are termed as the metal resistant micro-organisms. Most commonly, this resistance occurs *via* interaction of cationic metal ions with anionic cellular components, such as cell membrane, proteins, and DNA, and subsequent reduction, hydrolysis, or oxidation leads to the formation of metal, metal oxide, or other types of NPs. Many of the metal resistant micro-organisms have been used successfully for the nanoparticle synthesis. However, there remain major drawbacks associated with their implication which includes lack of homogeneity in terms of size and shape of these NPs and issues concerned with scaling up of this process in the industries. This demands the use of novel approaches involving biotechnology, molecular biology, and genetics to address these issues. The use of such engineered NPs has found applications in diverse areas ranging from cosmetics to medicine (Raj et al., 2012; Valavanidis and Vlachogianni, 2016). Genetic engineering allows for the tailor made synthesis of NPs using various microbes that are better suited for different applications. Various genetic engineering approaches used for the nanomaterial synthesis and assembly have been summarized in Figure 6.

### Use of Engineered Biological Scaffolds

The use of biological scaffold system has been used over the years to facilitate the biomineralization of metal ions leading to synthesis of nanomaterials. Expressing a recombinant virus scaffold system in bacteria has been shown to provide an endogenous system that can have a direct control over the size, shape, and phase of NPs during the nucleation stage. This has enabled the use of this virus-based toolkit to direct the synthesis of magnetic and semiconducting nanostructures. Phage display method has been used to identify phage proteins having substrate specificity toward NPs, control over its nucleation, and inherent ability to orient themselves as per the symmetry of phage capsid (Whaley et al., 2000; Lee et al., 2002; Mao et al., 2003). Expressing a genetically modified M13 Phage scaffold harboring peptides controlling the properties of nanostructures directs the synthesis of tailor made semiconducting and magnetic nanowires inside hosts. Since these proteins are genetically encoded and contained within capsid, multiple copies of it can be produced upon infection of a host bacterium (Mao et al., 2004). In another study, expressing recombinant noninfectious p22 viral cage proteins into a host, allows for the biomineralization of Fe_2_O_3_ NPs. While the inner capsid layer proteins initiate the nucleation of NPs, the outer shell proteins regulate the size of the NPs by providing a physical constrain. Additionally, homogeneity in nanoparticle size can be achieved by expressing polyanionic peptides that interact with capsid. The assembly of this viral cage can be easily altered using chemical, molecular, or genetic ways, thus giving a new level of control over the area of nanoparticle synthesis (Reichhardt et al., 2011).

In addition to the protein scaffold, expression of cell free DNA scaffold can be used to direct the assembly of cellular biosynthetic machinery. This DNA scaffold can then be used toward the synthesis of numerous metabolic products (Conrado et al., 2012). The DNA scaffold also directs the nucleation of metal NPs based on electrostatic interactions. The circular plasmid DNA from *Bacillus* spp. form stable complexes with Ag ions using charge-charge interactions. The negatively charged sugar phosphate backbone of plasmid DNA interacts with the positively charged Ag ions leading to stabilization and reduction into Ag nanoassemblies. The DNA-Ag ions complex formation is confirmed by measuring the absorbance of the solution at 420 nm, characteristic of Ag NPs. The control solution lacking the plasmid DNA but with Ag ions failed to give the characteristic absorbance confirming the role of plasmid DNA in reducing Ag ions to form NPs. This process is accelerated using the method of photo irradiation (Liu et al., 2012). Since plasmids can be isolated and purified from many bacteria on large scale, this method provides a promising future for the directed fabrication of nanomaterials.

### Expression of Metal Binding Proteins

Additionally, expression of metal binding proteins with affinity for heavy metals in foreign host has been proved to be effective for heavy metal removal. The first of this kind of study was performed with recombinant *E. coli* expressing phytochelatin synthase gene from *Schizosaccharomyces pombe* in addition to modified γ-glutamylcysteine synthetase which succeded in producing Cd nanocrystals (Kang et al., 2007). γ-Glutamylcysteine synthetase acts as a catalyst to the synthesis of glutathione which is a precursor of phycochelatin. Phycochelatin is a metal binding protein that acts as the capping agent for the nanostructures. Recombinant *E. coli* harboring genes for phycochelatin synthase from *Arabidopsis thaliana* and metallothionein from *Pseudomonas putida* produces NPs of various metals such as semi conducting, alkali earth, magnetic, para magnetic, noble, and rare earth fluorides. It was shown that by changing the concentration of metal ions, NPs of desired size could be obtained. Using the same system of recombinant *E. coli*, another group of researchers have been successful in synthesizing around 60 different types of nanomaterials that include 34 elements from the periodic table. Thus, recombinant *E. coli* expressing metal binding proteins can be used as a cellular factory for the synthesis of diverse NPs (Park et al., 2010; Choi et al., 2018).

Mammalian cells expressing recombinant protein that sequester iron NPs have made it easy for their MRI. The use of genetically engineered mammalian cells expressing recombinant protein system encapsulin/cargo from *Quasibacillus thermotolerans* along with encapsulin from *M. xanthus* provides a highly efficient genetic reporter system. Encapsulin is a family of iron sequestering proteins naturally that occurs in bacteria and archea. Upon expression in HEK293T cells, this encapsulin protein system self-assemble into nanospheres having icosahedral symmetry that further sequesters and encapsulates ferritin like proteins into it. The mammalian cells harboring this sequestered cargo protein enables its multiplexed gene reporter imaging using the conventional transmission electron microscopy (Sigmund et al., 2019). In magnetotactic bacteria, *magA* gene encodes for a protein involved in iron sequesteration and transport into the cells. Expressing this gene into mouse neuroblastoma cells N2A, causes iron loading and its conversion into NPs by cellular enzymes. This stabilized nanoparticle now provides a contrast signal system during the MRI that can be easily made nonfunctional with a single point mutation (Goldhawk et al., 2009).

### Fabrication of Magnetosomes

Till date, importance of individual genes in the synthesis of NPs has been studied using either gene knockdown or knock in approach. In the gene knockdown approach, the expression of a gene product is silenced using siRNA/miRNA and while in gene knock in approach, a gene or cluster of genes is introduced into a cell lacking it. In both cases, the impact of gene silencing or gene introduction on the rate of nanoparticle synthesis, properties of resulting NPs and their efficiency/suitability for various applications is observed (Das et al., 2016; Chen et al., 2019). Magnetosomes are the very finely ordered nanostructures found in magnetotactic bacteria that are membrane bound. These bacteria that are generally found in deep oceans use magnetosomes to align along the earth’s gravitational field. Many researchers are trying to elucidate the mechanistic pathway underlying the synthesis of these tiny magnetic structures as they can be exploited for biotechnology and nanotechnology purposes. However, they are limited owing to the fact that these bacteria are difficult to isolate from the deep ocean samples and also difficult to handle under laboratory conditions due to their fastidious nature (Stoller et al., 2020). Furthermore, the synthesis of magnetosomes is a very tidy process controlled by a complex set of genetic components (Jogler and Schuler, 2009). In 2001, a group of researchers observed a large cluster of genes conserved in several species of magnetotactic bacteria to be encoding proteins directly involved in the magnetosome formation (Grunberg et al., 2001). Later, the role of this conserved genetic loci termed as Magnetosome Island (MAI) was eludicated by studying the effect of deletion of individual genes from the island on the magnetization property (Murat et al., 2010). Earlier, it was shown that an acidic protein encoded by *mamJ* aligns the magnetosomes along a filament like structure and deletion of these genes affects the orientation of magnetosomes along earth’s magnetic field (Scheffel et al., 2006). In a breakthrough study, Kolinko et al. (2014) showed that the ability to mineralize these magnetic nanocrystals can be transferred to a heterologous host such as *E. coli via* transfer of a set of genes. The *mamAB* operon in *Magnetospirillum gryphiswaldense* encodes for proteins that are involved in the formation of magnetosome membrane, transport of iron into magnetosomes, crystallization of magnetite (Fe_3_O_4_) NPs and their orderly arrangement, and positioning inside the magnetosomes. The smaller operons, namely *mamGFDC*, *mms6*, and *mamXY*, play accessory roles in this process of biomineralization. Using a phage vector, a set of 29 genes from these four operons was stitched together in an expression cassette and introduced into photosynthetic bacterium *Rhodospirillum rubrum*. This study opened a new era of tailor made optimized synthesis of magnetic nanostructures in recombinant host of interest using synthetic biology approaches. Another study shows the importance of redox control mechanism along with carbon metabolism and iron supply in inducing synthetic bio magnetization in model organism *S. cerevisiae*. Screening of knockout mutants for inducing magnetization identified TCO89, a component of target of rapamycin complex 1 (TORC1) along with several genes for carbon metabolism in yeast as essential in inducing the bio magnetization (Nishida and Silver, 2012).

Recently, a group of researchers achieved genetic control over the physico-chemical properties of Fe_2_O_3_ NPs in *Magnetospirillum magneticum* AMB1. By fine tuning of ribosome binding sites, constitutive promoters, and use of inducible genetic system over a wide range, the size, shape, surface properties, and chain length of Fe_3_O_4_ NPs can be controlled (Furubayashi et al., 2020). This process is much more complicated than it appears due to multidimensional complexity associated with gene expression systems.

### Synthetic Gene Circuits

Recently, synthetic gene circuits are being designed and expressed to have a better control over the synthesis and properties of NPs made. For constructing a gene expression circuit, all the molecular components need to be assembled, the information of which is encoded in DNA itself. DNA binding enzymes can decode this information along with other enzymes that can catalyze the chemical reactions leading to metal binding. Some proteins contain region that bind metal ions and reduce them with the help of enzymes. Thus, multiple structural and enzymatic components are encoded by this synthetic gene circuits regulating the nanoparticle synthesis (Rice and Ruder, 2013). According to the central dogma of biology, DNA is transcribed into RNA by RNA polymerase and this RNA then binds to ribosomes to be translated into functional proteins. In the transcription step, binding of RNA polymerase to the promoter of specific DNA sequence results in the synthesis of complementary RNA sequence. This step requires precision and high specificity of RNA polymerase as any deviation from this will result in the expression of unwanted gene(s). Two types of proteins, activators, and repressors each having affinity for DNA decides the fate of this step. Activators can catalyze the binding of RNA polymerase to DNA promoter, while repressors can prevent this binding by itself binding to DNA. Similarly, in the second step, the sequence of RNA transcribed influences its binding to ribosomes. Using genetic engineering approaches, these sequences can be altered in precise and specified manner leading to control over gene expression. In 19th century, continuous efforts were made in modifying and optimizing the naturally occurring promoters to provide better control over the gene expression events. Several of bacterial and viral promoters were modified that responded to activators *AraC* and repressors *lacI* and *tetR* (Lutz and Bujard, 1997). The first synthetic promoter toggle designed. In 19th century consisted of two mutually repressing gene operons. When both the operons repress each other equally, the gene circuit is said to be bistable. This ability of repressing each other depends on many variables such as affinity and binding strength of DNA and RNA polymerases to DNA promoters, as well as the binding strength of ribosomes and its corresponding binding sites in mRNA. By altering the bases of each of these regions, the toggle switch can be turned ON or OFF even in the absence of inducer such as tetracycline and lactose (Gardner et al., 2000).

Another group of researchers designed AND gate in bacteria that consisted of genetically engineered RNA polymerase with a genetic defect in the genetic code that blocks its complete translation. Providing one input, for example, Arabinose results in partial translation of the protein while another input, example salicyclate results in restoration of this genetic defect to induce the expression of wild type full length RNA polymerase (Anderson et al., 2007). Using this approach, the fermentation timings could be controlled using genetically engineered strains of *S. cerevisiae* expressing synthetic timers (Ellis et al., 2009).

Recently, synthetic genetic circuits have been used to induce the bio sensing ability inside the biofilms. Biofilms provide a tremendous platform for the synthesis of nanostructures which can be controlled using synthetic gene circuits. Curli fibers are amyloid nanofibers synthesized by many species of *Enterobacteriae* and are responsible for community type behavior inside biofilms (Tursi and Tukel, 2018). The curli fiber consists of two subunits CsgA, the major subunit of the developing curli fibers, and CsgB, the minor curli subunit. The function of CsgB is to regulate the nucleation of CsgA on the cell wall of bacteria and its further assembly into nanofibers (Barnhart and Chapman, 2006). These fibers can provide a conductive surface which can be used to synthesize nanomaterials. Therefore, attempts are being made to increase metal binding capacity of these nanofibers so that they can accelerate the selective synthesis of NPs inside the biofilms. Recently, the gene sequence encoding CsgA was fused with another sequences encoding for short peptides. This synthetic gene circuit now encodes for modified curli nanofibers with specific binding affinity for precursor metal ions. These modified curli nanofibers now act as template to direct the synthesis of other metal nanostructures inside biofilms (Olmez et al., 2019).

The synthetic gene circuit has also been used to detect the cellular toxicity of nanomaterials. A synthetic gene circuit consisting of four heat shock promoter regions along with a gene sequence coding for reporter protein acts as the biosensor for the nanoparticle induce toxicity (Saltepe et al., 2019). Such synthetic biosensor circuit based on use of HSP system can be applied to assess the toxicity of other types of nanomaterials as well.

### Genetically Engineered Bacteriophages

Viruses are at the crossroads of living and non-living as they possess the genetic material necessary for all the living organisms but lack the machinery to make functional proteins. For this purpose, they rely on their host hence are referred to as obligate intracellular parasites. Upon injection of viral nucleic acid into host, multiple copies of it are produced using cellular polymerases. This is followed by transcription and translation of structural and functional viral proteins. The most important step of virus life cycle is the accurate assembly of viral capsid enclosing the nucleic acid to give rise to a functional virion. This process occurs *via* self-assembly of structural proteins into capsid having a defined symmetry (Koonin and Starokadomskyy, 2016). For long time, scientists are intrigued with this tremendous ability of viruses to self-assemble into nanomolecular assemblies and, thus, it has remained a topic for research over the years. Recently, researchers are using this ability of viral capsid to self-assemble for inducing the synthesis of nanomaterials having exotic applications.

The self-assembled nanoassembly of viruses has found applications as filters, sensors, photonics, and bio mimetics. Using the genetically engineered M13 bacteriophage, a nanoporous self-assembled biofilm structure is formed that filters selective ions from the sodium chloride (NaCl) solution. This self-assembled biofilm is multilayered with a pore diameter of 150–200 nm and a depth of 15–20 nm. It consists of alternating units of M13 phage along with polydiallyldimethylammonium chloride (PDDA) formed by pulling in and out method. The role of PDDA is to reduce the surface roughness of M13 phage allowing, thus facilitating the smooth assembly of biofilm. Using genetic engineering tools, negative charge of the bacteriophage is increased by inducing the expression of glumatic acid. Using this biofilm, Na^2+^ ions are trapped in phage layers, while Cl^−^ ions are trapped in PDDA layers (Devaraj et al., 2018). This method provides an eco-friendly, inexpensive, and energy efficient approach for selective fabrication of nanoassemblies for diverse applications. M13 bacteriophages have a small size and thus small genome and have a unique self-assembling property that leads to the formation of variety of nanostructures (Sawada and Serizawa, 2018).

Over last many years, bacteriophages have attracted attention of researchers worldwide due to their potential use as antimicrobial agent. Many research groups have successfully used this method to reduce bacterial burdens in different settings owing to the high degree of specificity displayed by phages toward their host bacterium. However, there are certain limitations on the *in vivo* usage of this therapy due to the poor control over the replication and spread of phages post clearance of bacterial infection and also the chances of facilitating horizontal gene exchange *via* the process of transduction (Principi et al., 2019; Brives and Pourraz, 2020). This problem can be overcome by coupling phages to Au nanorods, thereafter called as phanorods that can be destroyed upon photothermal heating after its use. The chimeric phages expressing increased specificity, specifically target the bacterial cells. Subsequent photothermal ablation leads to destruction of both host cells as well as bacterial cells. The Au nanorods conjugated phages can be excited using near infra-red light. Upon excitation, they emit energy *via* a process of non-radioactive decay which leads to the generation of heat in targeted bacterial cells leading to their destruction (Peng et al., 2020).

The nucleation and size of nanostructures has been shown to be controlled by peptides. Therefore, bacteriophages expressing these peptides upon exposure to the chemical precursor molecules initiate the accumulation and crystallization of corresponding nanostructures on the phage capsid (Karimi et al., 2016). The specificity displayed by bacteriophages toward host bacteria is executed through the highly specific protein-protein interaction between phages and bacteria. Therefore, expressing this phage protein rather than using the whole phage seems to provide an alternative, at least, for few applications. The tail fibers and spikes of phages express receptor binding proteins (RBPs) by which they bind to complementary cell wall proteins of their host bacterium (Dunne et al., 2019). Expressing these RBPs has found to be useful for the detection of some of the human pathogens such as *Salmonella*, *Shigella*, and *Pseudomonas* spp. (Schmidt et al., 2016; He et al., 2018; Kunstmann et al., 2018). Since a long time, TMV has been used for application oriented synthesis of NPs. These viruses can be modified to flank metal binding proteins/peptides to direct the controlled synthesis of Au NPs of uniform size and crystalline shape. Two short amino acid motifs, GASL and SEKL, were found to stimulate the synthesis Ag nanoplates which are much bigger in size than other nanostructures. It was observed that repeating these sequences in the metal binding peptide being displayed on the phage capsid accelerates this process upon incubation with Ag salts (Love et al., 2015). These peptides may act as the biological catalyst for the synthesis of these nanostructures. Use of reduced aromatic amino acids such as tryptophan in combination with these repeating sequences further speed up the process of Ag nanoplate formation (Tan et al., 2010). Genetically modified phages can be used for reduction of metals into nanostructures even in the absence of reducing chemical agents. Using reducing biological compounds such as dipeptide consisting of dual cysteine residues, modified TMV was shown to synthesize Pd NPs at temperatures above 50°C (Lim et al., 2010).

### Genetic Tuning of Quantum Dots

Quantum dots (QDs) are luminiscent NPs that has revolutionized the field of nanotechnology because of its unparalleled importance in biomedical fields and diagnostics. They are made up of semiconducting material that has unique electronic and optical characteristics and can also transfer electrons. A typical QD consists of a core made of semiconducting material, a shell made of metallic layer such as ZnS to enhance the optical properties and a cap to increase the solvent accessibility. Several types of QDs have been synthesized by now are made up of hetero nano-structures such as CdS, PbSe, CdTe, ZnS, and PbS. These QDs range in size from 1 to 10 nm and exhibit quantum effect owing to the quantum confinement.

The breakthrough study for the biosynthesis of Cds QDs was performed in 1989 using yeasts species such as *S. pombe* and *Candida glabrata*. These species accumulated CdS crystallites in the presence of Cd salts. A short peptide was used to control the nucleation and the growth of these nanocrystals. The nanocrystals synthesized by this green approach were uniform in size (monodisperse) compared to those synthesized chemically (Dameron et al., 1989). This approach was later used in multiple micro-organisms enabling the efficient synthesis of QDs. Using genetically engineered *E. coli* expressing a binding peptide, CdS nanocrystals were synthesized that are more compatible for bio imaging and labeling (Mi et al., 2011). In a similar study, *gshA* gene involved in glutathione biosynthesis was over expressed in *E. coli*. Upon exposure to CdCl_2_ and K_2_TeO_3_ salts, these engineered *E. coli* cells accumulated increased levels of CdTe hetero nanostructures compared to the wild type bacteria. Overexpression of *gshA* gene increases the cellular content of reduced thiol compounds leading to reduction of metals ions (Monras et al., 2012).

In addition to the genetic control over QD synthesis, a novel approach involves coupling the QD synthetic pathways to non-related biochemical reaction. This novel approach has been demonstrated successfully for the synthesis of CdSe QDs using yeast as a model organism. The biosynthesis of CdSe QDs usually occurs at higher temperature (300°) using the toxic solvents (Seo and Kim, 2007; Ratnesh and Mehata, 2015). By using the biogenic approach, the reaction temperature can be reduced significantly also providing the control over the photoluminiscent properties of CdSe nanocrystals. Upon exposure, yeast cells take up the selenite and convert it into glutathione selenotrisulfide with the help of reduced glutathione (GSH) and GSH related enzymes such as NADPH and glutathione reductase. Since this conversion depends on growth phase, exposing the stationary phase yeast cells to selenite salts maximizes its conversion into organoselenium compounds such as selenocysteine and selenomethionine. After this, co-incubating these seleniumized yeast cells to Cd salts at appropriate time leads to accumulation to CdSe QDs which can be easily observed using fluorescence microscopy. Thus, coupling metabolic pathways in space and time can be used as an efficient strategy to exert control over the synthesis of QDs (Cui et al., 2009). The importance of glutathione metabolic pathway was highlighted by studies involving deletion of several genes of these pathways. Deletion of *gsh1* and *gsh2* genes controlling the first and second stages of glutathione biosynthetic pathway, respectively, lead to the decrease in the fluorescence intensity of intracellular CdSe QDs. Addition of CdCl_2_ to selenumized yeast cells showed 8-fold increase in the expression of *gsh1* gene using RT-PCR which further highlighted its role in CdSe biosynthetic pathway. Deletion of another gene, *glr* which increases the level of oxidized glutathione (a chemical analog of glutathione) also cannot restore the reduced ability of yeast cells to synthesize CdSe nanocrystals. However, addition of exogenous glutathione restored this ability to a great extent (Li et al., 2013). This study strengthened the role of glutathione synthesis pathway in the intracellular formation of CdSe QDs. The route involving the intracellular formation of CdSe QDs has also been applied toward the directional synthesis of other hetero nanostructures such as Au NPs, Au-Ag alloy NPs, Au clusters, PbSe nanocubes, and Ag_2_Se QDs (Cui et al., 2010, 2012; Zhang et al., 2011; Gu et al., 2012). This implies the significant importance of this biosynthetic route in tailored biogenesis of nanostructures in other microorganisms.

## Conclusion

The review represents the impact of a combination of genetic engineering, biotechnology, microbiology, molecular biology, synthetic biology, and metabolic engineering approaches on the tailor made NP synthesis to have better functional properties. With the increasing demand of these nanostructures, technological advancements have been made to facilitate the customized application oriented synthesis of NPs. However, the increasing usage of these nanostructures also poses a bigger risk of harming the ecosystem which in long run would have unimaginable detrimental effects. Therefore, a balance needs to be maintained between the ecosystem maintenance and technological advancements.

## Future Prospects and Challenges

Beyond this review highlighting the advancements in the field of microbial nanotechnology, further research is essential for the widespread applications of nanomedicine while preserving the ecosystem. The concept of controlled drug release at target sites, bio distribution of these drugs and their intended effects at the tissue/cellular level, as well as theoretical mathematical prediction models yet need to be improved. Many studies in nanomedicine field focus on biomedical and formulation studies that appear in the early stages of biomedical applications. Therefore, it is essential to implement these approaches using *in vivo* models to further enhance its feasibility toward human welfare. To date, biological synthesis of metal NPs has been performed primarily at the laboratory scale. Hence, industrial scale adaptation is necessary for mass production. With appropriate optimized conditions and suitable micro-organisms, these “bio-nano-factories” can produce stable NPs with well-defined shapes, structures, and morphologies. Appropriate business strategies should result in the creation of a non-toxic biological system capable of producing metal NPs which will be another milestone toward sustainable development. Vaccines containing NPs have generated much interest in recent years, and a wide variety of NPs have been developed and used as delivery vehicles or immune enhancers. This approach has not only improved the antigen stability, antigen processing, and immunogenicity, but also leads to targeted delivery and slow release of antigens. In addition, NPs are not only used as antigens of interest but also as adjuvants in vaccine preparations. Till date, the available literature on synthesis of NPs shows heterogeneity in size with some actually measuring in nanometers while others measured in submicrons (more than 100 nm). Therefore, in depth understanding of the factors that govern the size and shape of NPs is essential to achieve consistent homogeneity. Other parameters which need more attention are the drug loading and release capacity of these NPs.

Nowadays, *de novo* protein engineering and *in silico* techniques have developed rapidly and can play an important role in supramolecular nanomaterial engineering for specific applications. Several challenges remain, including the difficulty of synthesizing non-aggregating NPs that have coherent and desirable properties, a fundamental lack of understanding of how the physical properties of NPs affect their bio-distribution and orientation, and how these properties affect their interactions with the biological system at different levels from cell to the tissue and organ. Other state-of-the-art techniques, such as computational design, material genomes, and artificial intelligence, can be integrated to discover more effective and translational NPs based on bioengineering strategies.

With better control over nanomaterials synthesis, researchers can make the nanomaterial world more amazing. However, current synthesis technology remains bottleneck that prevents in-depth exploration of the properties and applications of nanomaterials. Therefore, there is a lot of scope for improvement. There has been great enthusiasm for the simple approach toward the development of nano-robots (and nano devices) acting in tissue diagnosis, drug delivery, combating deadly virus like *SARS*, *Ebola*, and *Covid-19*, and repair mechanisms with complete external control mechanisms. This is not yet a reality and is still a future investigation that perhaps humanity can achieve in the very near future.

However, along with its benefits, the potential risk of nanomedicine toward both humans and the environment also needs long-term studies. Since nanomedicine has revolutionized the field of drug discovery and its administration in biological systems, the need for the regulation over its usage in healthcare and environmental systems is also increasing. Therefore, the appropriate acute or chronic toxicity effects of new nanomaterials on humans and the environment should be analyzed appropriately. Finally, the disposal of such nanomaterials into environment should be dealt with strict guidelines and regulations.

## Author Contributions

RD, TH, PT, and SK wrote the manuscript. SK and TH conceived the idea. SK compiled the manuscript. KS made the illustrations. All authors contributed to the article and approved the submitted version.

### Conflict of Interest

The authors declare that the research was conducted in the absence of any commercial or financial relationships that could be construed as a potential conflict of interest.

## Figures and Tables

**Table 1:** 

Ag	Silver
Ag_2_Se	Silver selenide
AIDS	Acquired immunodeficiency syndrome
Al_2_O_3_	Aluminium oxide
Au	Gold
AuCl4	Tetrachloroaurate
BMV	Brome mosaic virus
CaCO_3_	Calcium carbonate
CCMV	Cowpea chlorotic mottle virus
CdCl_2_	Cadmium chloride
CdS	Cadmium sulfate
CdSe	Cadmium selenide
CdTe	Cadmium telluride
CFB	*Cytophaga/flexibacter/bacteroides*
Co	Cobalt
CO_2_	Carbon dioxide
COVID-19	Coronavirus disease 2019
Cr	Chromium
Cu	Copper
Cyb5R	Cytochrome b5 reductase enzyme
DNA	Deoxyribose nucleic acid
EPS	Extra cellular polysaccharides
Fe	Iron
Fe_2_O_3_	Iron oxide
Fe_3_O_4_	Ferrosoferric oxide/magnetite
Fe_3_S_4_	Greigite
FISH-PAN	Fluorescent *in situ* hybridization using peptide nucleic acid probes
GSH	Glutathione
HBV	Hepatitis B virus
ICG	Indocyanine green
K_2_TeO_3_	Potassium tellurite
Li	Lithium
MAI	Magnetosome Island
MB	Methylene blue
Mn	Manganese
MRG	Metal resistance genes
MRI	Magnetic resonance imaging
Na_2_SeO_3_	Sodium selenite
NaCl	Sodium chloride
Ni	Nickel
NIR	Near-infrared
4-NP	4-Nitrophenol
NPs	Nanoparticles
PbS	Lead sulfide
PbSe	Lead selenide
Pd	Palladium
PDDA	Polydiallyldimethylammonium chloride
Pt	Platinum
QDs	Quantum dots
RBPs	Receptor binding proteins
RCNMV	Red clover necrotic mosaic virus
Rh	Rhodium
RNA	Ribonucleic acid
ROS	Reactive oxygen species
RT-PCR	Real time polymerase chain reaction
Ru	Ruthenium
S-layer	Surface layer
SARS	Severe acute respiratory syndrome
Sb_2_O_3_	Antimony trioxide
Se	Selenium
SiO_2_	Silicon dioxide
SRB	Sulfur reducing bacteria
TR	Translational repression
T4P	Type IV pili
TEM	Transmission electron microscopy
TiO_2_	Titanium dioxide
TMV	Tobacco mossaic virus
TORC1	Target of rapamycin complex 1
TR	Translational repression
U	Uranium
ZnO	Zinc oxide
ZnS	Zinc sulphide
